# Detection of SARS-CoV-2 by Use of the Cepheid Xpert Xpress SARS-CoV-2 and Roche cobas SARS-CoV-2 Assays

**DOI:** 10.1128/JCM.00772-20

**Published:** 2020-07-23

**Authors:** Angelica Moran, Kathleen G. Beavis, Scott M. Matushek, Carol Ciaglia, Nina Francois, Vera Tesic, Nedra Love

**Affiliations:** a Department of Pathology, The University of Chicago, Chicago, Illinois, USA; b Clinical Microbiology Laboratory, University of Chicago Medicine, Chicago, Illinois, USA; Boston Children's Hospital

**Keywords:** COVID-19, Cepheid Xpert Xpress SARS-CoV-2, Roche cobas SARS-CoV-2 assay, SARS-CoV-2

## LETTER

Severe acute respiratory syndrome coronavirus 2 (SARS-CoV-2), a novel coronavirus responsible for a December 2019 outbreak in Wuhan, China, causes a syndrome characterized by fever, cough, and dyspnea progressing to acute respiratory distress syndrome (1). SARS-CoV-2 quickly spread to other countries, with the new coronavirus disease 2019 (COVID-19) declared a pandemic in March 2020 (2–4). Rapid testing for SARS-CoV-2 is important for epidemiological tracking and institution of quarantine procedures (5). The clinical description of COVID-19 continues to evolve; with transmission by asymptomatic individuals reported (6–8), widespread testing is necessary.

Multiple reverse transcription-PCR (RT-PCR) assays have received emergency use authorization from the U.S. Food and Drug Administration. The Roche cobas SARS-CoV-2 assay is a qualitative test that detects SARS-CoV-2-specific ORF1 and part of the E gene, conserved across sarbecoviruses, including SARS-CoV-2 (9). The Cepheid Xpert Xpress SARS-CoV-2 assay also detects the pan-sarbecovirus E gene but detects the N2 region of the N gene as its SARS-CoV-2-specific target (10). This report compares results from specimens tested with both assays.

Eight nasal and 95 nasopharyngeal specimens were collected from inpatients and ambulatory patients at the University of Chicago. Samples were tested by the Roche cobas SARS-CoV-2 assay on the cobas 6800 system (Roche Molecular Systems, Branchburg, NJ) and by the Cepheid Xpert Xpress SARS-CoV-2 assay on the GeneXpert system (Cepheid, Sunnyvale, CA). Of these 103 specimens, 42 tested positive and 60 tested negative with both systems, for an agreement of 99%. Testing was repeated on the single specimen with discrepant results. For this specimen, the Roche assay was repeatedly negative for SARS-CoV-2. The initial Cepheid assay result was positive for SARS-CoV-2, though the cycle threshold (*C_T_*) values for detection of the E gene were 0.0 (negative) and 42.0 (low positivity) for the N gene. Repeat Cepheid testing was negative for both targets. These results suggest that SARS-CoV-2 was present at a very low concentration, near the detection limit of the Cepheid assay.

For the 42 positive samples, *C_T_* values for the E gene ranged from 15.05 to 39.75 (mean, 26.35; standard deviation [SD], 6.69) for the Roche assay and 13.6 to 38.2 (mean, 24.8; SD, 7.1) for the Cepheid assay. By Bland-Altman analysis to assess agreement, *C_T_* values were lower in the Cepheid assay for 37 of 42 samples (mean difference, –1.57; 95% limits of agreement, –5.34, 2.20). This might be due to differences in primer sequences for the E gene, reagents, or amplification conditions.

Limitations of this study include the small sample size of SARS-CoV-2-positive specimens, as testing was limited to patients within our institution. The assays also detect different SARS-CoV-2-specific genes, which may lead to false-negative results if a mutation prevents primer binding. The Cepheid assay is a 45-min random-access assay, with throughput dependent on the number of instrument slots. The Roche platform is batch based, accommodating 90 samples/run every 90 min. As each run requires up to 3 h and 45 min, throughput is approximately 1 result per minute. Overall, the Cepheid Xpert Xpress SARS-CoV-2 and Roche cobas SARS-CoV-2 assays show excellent agreement (>99%), and their combined usage can be tailored to maximize SARS-CoV-2 testing.

## References

[1] Chen N, Zhou M, Dong X, Qu J, Gong F, Han Y, Qiu Y, Wang J, Liu Y, Wei Y, Xia J, Yu T, Zhang X, Zhang L 2020 Epidemiological and clinical characteristics of 99 cases of 2019 novel coronavirus pneumonia in Wuhan, China: a descriptive study. Lancet 395:507–513. doi:10.1016/S0140-6736(20)30211-7. 32007143 PMC7135076

[2] Ki M, Task Force for 2019-nCoV. 2020 Epidemiologic characteristics of early cases with 2019 novel coronavirus (2019-nCoV) disease in Korea. Epidemiol Health 42:e2020007. doi:10.4178/epih.e2020007. 32035431 PMC7285424

[3] Spiteri G, Fielding J, Diercke M, Campese C, Enouf V, Gaymard A, Bella A, Sognamiglio P, Sierra Moros MJ, Riutort AN, Demina YV, Mahieu R, Broas M, Bengner M, Buda S, Schilling J, Filleul L, Lepoutre A, Saura C, Mailles A, Levy-Bruhl D, Coignard B, Bernard-Stoecklin S, Behillil S, van der Werf S, Valette M, Lina B, Riccardo F, Nicastri E, Casas I, Larrauri A, Salom Castell M, Pozo F, Maksyutov RA, Martin C, Van Ranst M, Bossuyt N, Siira L, Sane J, Tegmark-Wisell K, Palmerus M, Broberg EK, Beaute J, Jorgensen P, Bundle N, Pereyaslov D, Adlhoch C, Pukkila J, Pebody R, Olsen S, Ciancio BC 2020 First cases of coronavirus disease 2019 (COVID-19) in the WHO European Region, 24 January to 21 February 2020. Euro Surveill 25:2000178. doi:10.2807/1560-7917.ES.2020.25.9.2000178. PMC7068164 32156327

[4] Holshue ML, DeBolt C, Lindquist S, Lofy KH, Wiesman J, Bruce H, Spitters C, Ericson K, Wilkerson S, Tural A, Diaz G, Cohn A, Fox L, Patel A, Gerber SI, Kim L, Tong S, Lu X, Lindstrom S, Pallansch MA, Weldon WC, Biggs HM, Uyeki TM, Pillai SK, Washington State 2019-nCoV Case Investigation Team. 2020 First case of 2019 novel coronavirus in the United States. N Engl J Med 382:929–936. doi:10.1056/NEJMoa2001191. 32004427 PMC7092802

[5] Deng SQ, Peng HJ 2020 Characteristics of and public health responses to the coronavirus disease 2019 outbreak in China. J Clin Med 9:E575. doi:10.3390/jcm9020575. 32093211 PMC7074453

[6] Bai Y, Yao L, Wei T, Tian F, Jin DY, Chen L, Wang M 2020 Presumed asymptomatic carrier transmission of COVID-19. JAMA 323:1406. doi:10.1001/jama.2020.2565. PMC7042844 32083643

[7] Rothe C, Schunk M, Sothmann P, Bretzel G, Froeschl G, Wallrauch C, Zimmer T, Thiel V, Janke C, Guggemos W, Seilmaier M, Drosten C, Vollmar P, Zwirglmaier K, Zange S, Wolfel R, Hoelscher M 2020 Transmission of 2019-nCoV infection from an asymptomatic contact in Germany. N Engl J Med 382:970–971. doi:10.1056/NEJMc2001468. 32003551 PMC7120970

[8] Borges do Nascimento IJ, Cacic N, Abdulazeem HM, von Groote TC, Jayarajah U, Weerasekara I, Esfahani MA, Civile VT, Marusic A, Jeroncic A, Carvas Junior N, Pericic TP, Zakarija-Grkovic I, Meirelles Guimaraes SM, Luigi Bragazzi N, Bjorklund M, Sofi-Mahmudi A, Altujjar M, Tian M, Arcani DMC, O’Mathuna DP, Marcolino MS 2020 Novel coronavirus infection (COVID-19) in humans: a scoping review and meta-analysis. J Clin Med 9:E941. doi:10.3390/jcm9040941. 32235486 PMC7230636

[9] Roche Molecular Systems. 2020 cobas SARS-CoV-2. (Package insert.) US Food and Drug Administration, Silver Spring, MD https://www.fda.gov/media/136049/download. Accessed 8 April 2020.

[10] Cepheid. 2020 Xpert Xpress SARS-CoV-2. (Package insert.) US Food and Drug Administration, Silver Spring, MD https://www.fda.gov/media/136314/download. Accessed 8 April 2020.

